# Anthraquinone laxatives use and colorectal cancer: A systematic review and meta‐analysis of observational studies

**DOI:** 10.1002/ptr.7373

**Published:** 2022-01-17

**Authors:** Niccolò Lombardi, Giada Crescioli, Valentina Maggini, Raffaele Bellezza, Iacopo Landi, Alessandra Bettiol, Francesca Menniti‐Ippolito, Ilaria Ippoliti, Gabriela Mazzanti, Annabella Vitalone, Eugenia Gallo, Francesco Sivelli, Francesco Sofi, Gian Franco Gensini, Alfredo Vannacci, Fabio Firenzuoli

**Affiliations:** ^1^ Department of Neurosciences, Psychology, Drug Research and Child Health, Section of Pharmacology and Toxicology University of Florence; Tuscan Regional Centre of Pharmacovigilance and Phytovigilance Florence Italy; ^2^ Tuscan Regional Centre of Pharmacovigilance Florence Italy; ^3^ Research and Innovation Center in Phytotherapy and Integrated Medicine CERFIT, Referring Center for Phytotherapy, Tuscany Region Careggi University Hospital Florence Italy; ^4^ Department of Experimental and Clinical Medicine University of Florence Florence Italy; ^5^ National Centre for Drug Research and Evaluation National Institute of Health Rome Italy; ^6^ Department of Physiology and Pharmacology “Vittorio Erspamer” Sapienza University of Rome Rome Italy; ^7^ IRCCS MultiMedica, Sesto San Giovanni Milan Italy

**Keywords:** anthraquinone, colorectal cancer, laxative, meta‐analysis, phytovigilance, systematic review

## Abstract

This systematic review and meta‐analysis were conducted to determine the effects of anthraquinone (AQ) laxatives on colorectal cancer (CRC). We searched PubMed, Embase, Google Scholar, and CENTRAL from inception until March 2021, for randomized controlled trials (RCTs) and observational studies. Through the systematic review, we identified 8 observational studies evaluating AQ laxatives use as a risk factor for CRC development, and 5 studies on CRC risk were included in the meta‐analysis using a random‐effects model. Through the meta‐analysis, we found that a history of AQ laxatives use compared with “other” and “no laxatives” use was associated with CRC development (OR: 1.41; 95% CI: 0.94–2.11), although not at a statistically significant level. The possible association persists even after removal of the outlier studies (OR: 1.51; 95% CI: 0.97–2.34). Selection of cases and controls was judged at low or unclear risk of bias across almost all studies, and the quality of evidence was from moderate to low. In conclusion, it is not possible to associate the use of AQ laxatives with the development of CRC. However, the trend toward an increased risk of CRC provides a strong indication for investigating this issue by performing further high‐quality studies.

## 1 INTRODUCTION

Anthraquinone (AQ) derivatives are a class of chemical substances naturally occurring in different botanical species and used in food to improve bowel function (Gordon, Macdonald, Parker, Akobeng, & Thomas, 2016), and for some of them there are contradictory data on a possible carcinogenic risk (van Gorkom, de Vries, Karrenbeld, & Kleibeuker, 1999).

The European Food Safety Authority (EFSA) reported that some AQ derivatives in food supplements represent a relevant health problem (Younes et al., 2018). The EFSA Panel on Food Additives and Nutrient Sources added to Food reviewed the available scientific evidence on a possible relationship between AQ derivatives exposure and carcinogenic effects. On the basis of the data currently available, the Panel noted that some derivatives (such as emodin, aloe‐emodin, and the structurally related substance danthron) showed evidence of in vitro and in vivo genotoxicity. Notably, the Panel was unable to provide advice on a health‐safe daily intake of AQ derivatives. However, EFSA discouraged the long‐term use and high‐dose consumption of AQ derivatives because of potential safety issues, in particular an increased risk for colorectal cancer (CRC) (Lombardi et al., 2020). Moreover, the use of AQ‐containing laxatives has been associated to the development of melanosis coli (MC), a clinical condition characterized by a black or brown pigment deposited in the colorectal mucosa (Yang, Ruan, & Jin, 2020), which is not a pre‐neoplastic lesion.

In March 2021, the European Commission confirmed the adoption of the regulation prohibiting the use of all preparations based on *Aloe* spp., as well as those containing emodin and aloe‐emodin, through the amendment of Annex III of Regulation (EC) no. 1925/2006 relating to botanical species containing AQ derivatives (European Commission, 2021). Furthermore, as there is a possibility of harmful effects on health associated with the use of *Rheum*, *Cassia*, and *Rhamnus* and their preparations in food supplements, such substances were placed under Union scrutiny and therefore, were included in Part C of Annex III to Regulation (EC) no. 1925/2006 (European Commission, 2006).

Products containing AQ derivatives are known to be used primarily worldwide as oral laxatives and have various biological effects (Lombardi et al., 2020), also associated with an increased risk of serious adverse events (AEs). In contrast, there is no clear qualitative and quantitative evidence on the association between their use and the risk of CRC onset. In this context, we conducted a systematic review of the literature and meta‐analysis to estimate the overall CRC risk associated with the use of oral laxatives‐containing AQ derivatives.

## 2 METHODS

### 2.1 Search strategy

This systematic review and meta‐analysis was conducted following the Preferred Reporting Items for Systematic Reviews and Meta‐Analyses, the Preferred Reporting Items for Systematic Reviews and Meta‐Analyses statement (Moher et al., 2009), and according to the protocol registered in PROSPERO (Registration Number: CRD42019125414) and published in January 2020 (Lombardi et al., 2020). A literature search was performed in PubMed (last search performed on March 18, 2021). The PubMed search strategy is available in the aforementioned published protocol (Lombardi et al., 2020). The PubMed search strategy was also adapted to the syntax and subject headings of the Embase. We also searched the online databases Google Scholar and CENTRAL. Records were retrieved on the same day from all sources and the search strategy was updated toward the end of the review, after being validated to ensure it retrieved a high proportion of eligible studies.

### 2.2 Inclusion/exclusion criteria

We considered for inclusion both randomized clinical trials and observational cohort studies, either prospective or retrospective. We also included case–control studies. Observational cross‐sectional studies, reviews and meta‐analyses, letters to the editor, case reports, case series, and expert opinions, were excluded. Subjects with no age restrictions and taking AQs as oral laxatives, excluding patients with history of any cancer, were considered. The following plant‐containing AQ laxatives were included: Senna, syn. Cassia (*Cassia acutifolia*, *C. angustifolia*); Frangula (*Rhamnus frangula*); Cascara (*Rhamnus purshiana*, *Syn. Cascara sagrada*); Rhubarb (*Rheum officinale*, *R. palmatum*); Aloe spp. (*Aloe vera*, *syn. A. barbadensis*, *A. ferox*, *A. arborescens*). We also considered all active AQ compounds, such as physcion, chrysophanol, rhein, dantron, emodin, aloe‐emodin, and senna glycosides (sennoside A and B). Studies on patients co‐treated with more than one aforementioned AQ laxatives were included, as well. We considered studies evaluating the effect of the aforementioned AQ laxatives compared to no treatment and/or compared to non‐AQ laxatives. There was no restriction by type of setting, and we included articles written in any language.

### 2.3 Study selection

Two review authors (NL and GC) have independently screened the extracted records and identified the studies for inclusion by screening titles and abstracts yielded by search, eliminating those deemed irrelevant. We retrieved full‐text articles for all references that at least one of two review authors identified for potential inclusion. We selected studies for inclusion on the basis of review of full‐text articles. Any discrepancy between the findings of two review authors was resolved through discussion.

### 2.4 Quality assessment

Two review authors (N.L. and V.M.) independently assessed the included studies for bias. To assess the risk of bias of included randomized controlled trials, we followed the Cochrane Handbook for Systematic Reviews of Interventions (Higgins et al., 2011). To assess the risk of bias of observational studies, we followed the Newcastle‐Ottawa Quality Assessment Scale (Wells et al., 2021). For each domain in the two tools, a judgment as to the possible risk of bias was made from the information reported in the body of papers, rating from “low‐risk” to “high‐risk.” The judgements were made independently by two review authors; disagreements were resolved first by discussion and then by consulting a third author. A graphic representation of potential bias will be provided, using the software RevMan 5.4.1 (Review Manager 5.4.1) (Cochrane Organisation, 2021).

### 2.5 Data extraction

Data were independently extracted from each article by two authors using a data collection form. Data was extracted at the trial arm level. Extracted data included the name of the study authors and year of publication, the study design and characteristics (including single or double blinding and randomization), and the country in which participants were recruited. As for the population, we extracted the subjects' age, and clinically relevant comorbidities. As for the intervention and the comparator, we extracted the active principle of the experimental intervention, its route of administration, the treatment dosage, and the duration of treatment. We extracted the number of patients recruited, the number of participants included in the analysis, the number of participants with events for binary outcomes, effect size measurements (i.e., odds ratio [OR]), and variables entering the multivariable model as potential confounders, if appropriate. We resolved discrepancies between the findings of two review authors through discussion.

### 2.6 Study outcomes

The primary and secondary safety outcomes were “CRC” and “MC,” respectively. In studies evaluating at least one of the aforementioned safety outcomes, we also considered the following AEs: gastrointestinal bleeding, alterations in gastrointestinal motility, and potential for dependence. Any AE, if present, was identified based on specific authors' definitions, and classified using the MedDRA classification, according to PT and SOC classification (“MedDRA Hierarchy–Medical Dictionary for Regulatory Activities” [MedDRA Hierarchy, n.d.]). Regarding the time of outcomes onset, we defined an oral consumption of AQ laxatives less than 2 weeks as “short‐term” use, while “long‐term” use will be referred to as oral consumption longer than 2 weeks.

### 2.7 Statistical analysis

All considered outcomes were based on dichotomous data. Heterogeneity was evaluated using the *I* ^2^ Higgins test (Ruppar, 2020). According to the assessment of statistical heterogeneity, we performed a meta‐analysis using a random‐effects model, calculating pooled ORs and related confidence intervals (CIs) combining the estimates reported in each study using random‐effects Mantel–Haenszel method. The proportions of each reported AE was described at study level. A *p value* less than .05 was considered statistically significant. Statistical analyses were conducted using STATA software, version16.1.

## 3 RESULTS

### 3.1 Study selection and characteristics

**FIGURE 1:**
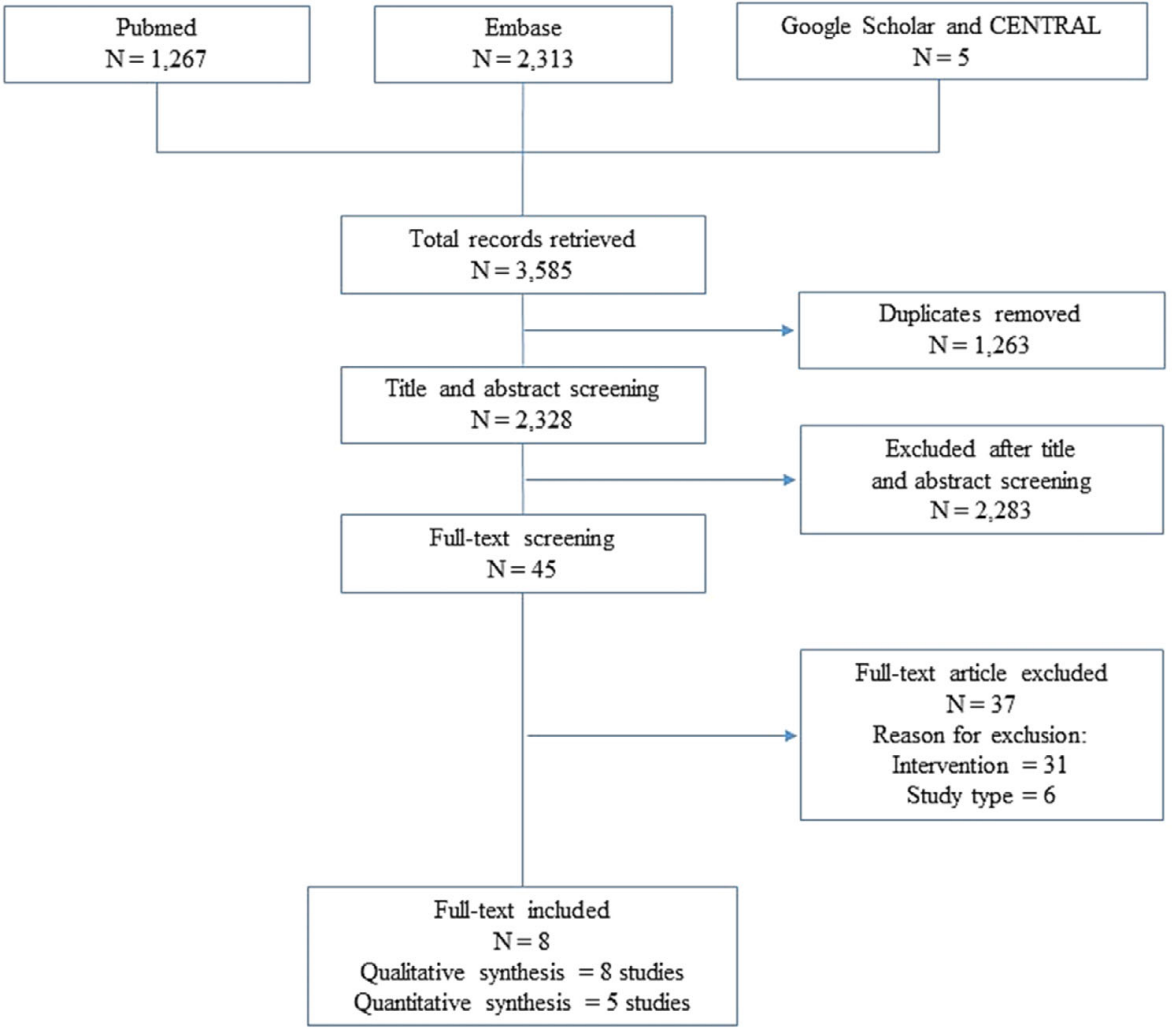
Systematic review and meta‐analysis diagram of searching

A total of 3,585 citations were identified through database searching. After removing duplicates (*n* = 1,263), 2,328 citations were screened, excluding 2,283 records by title and abstract. Forty‐five citations met inclusion criteria for full‐text review. Thirty‐one studies were excluded due to the intervention and 6 due to the study design (Figure 1). Eight manuscripts were finally included in the systematic review for a total of 41,873 patients. Of them, 5 were case–control (Badiali et al., 1985; Boyd & Doll, 1954; Kune, 1993; Nascimbeni et al., 2002; Nusko, Schneider, Schneider, Wittekind, & Hahn, 2000), and 3 were cohort studies (Nusko, Schneider, Ernst, Wittekind, & Hahn, 1997; Nusko, Schneider, Muller, Kusche, & Hahn, 1993), of which one presented a case–control nested design (Charlton, Snowball, Bloomfield, & De Vries, 2013) (Table 1). Four studies considered both CRC and MC (Nascimbeni et al., 2002; Nusko et al., 1993, 1997, 2000), three studies considered CRC only (Boyd & Doll, 1954; Charlton et al., 2013; Kune, 1993), while one study MC only (Badiali et al., 1985). The percentage of patients with a history of laxatives use across studies ranged from 12 to 100%. Although definitions of CRC and/or MC were homogenous, information on laxative exposure (in particular, dosage and treatment length) was lacking among the majority of included studies. Information regarding AEs were not reported in the included studies.

**TABLE 1:** Full‐text manuscripts included in the systematic review of colorectal cancer and/or melanosis coli and any laxatives use (*qualitative evidence*)

First author	Publication year	Years included	Study design	Country	Number of patients	Age	Treatments	Dosage	Treatment length	Outcome
Boyd & Doll	1954	1948–1949	Case–control	United Kingdom	1927	<45 years: 246 45–54 years: 563 55–64 years: 666 >65 years: 452	Any laxatives (*n* = 1,129)	Not reported	Continuous period of at least 5 years	Cancer of large bowel
							No laxatives (*n* = 798)			
Badiali et al.	1985	Not reported	Case–control	Italy	114	Melanosis: Median 38 (10–73) years; No melanosis: Median 40 (5–78) years	Any laxatives (*n* = 54)	Not reported	Not reported	Melanosis coli
							No laxatives (*n* = 14)			
Kune et al.	1993	1980–1981	Case–control	Australia	1,442	Mean 65 (*SD* +11) years	Any laxatives (*n* = 324)	Not reported	Not reported	Colorectal cancer
							No laxatives (*n* = 1,084)			
Nusko et al.	1993	1988–1991	Cohort, retrospective	Germany	2,277	Mean 52.9 (*SD* ±16.5) years	Any laxatives (*n* = 271)	Not reported	Not reported	Carcinomas of the large bowel, melanosis coli
							No laxatives (*n* = 2006)			
Nusko et al.	1997	1985–1992	Cohort, retrospective	Germany	2,229	Median 55 (range 30–80) years	Any laxatives (*n* = 372)	Not reported	Not reported	Colorectal carcinoma, melanosis coli
							No laxatives (*n* = 1857)			
Nusko et al.	2000	1993–1996	Case–control	Germany	554	Mean 58.47 years	Any laxatives (*n* = 97)	Not reported	Carcinoma group: Mean duration of anthranoid laxative 12.9 years; Control group: Mean duration of anthranoid laxative 14.8 years	Colorectal neoplasm, melanosis coli
							No laxatives (*n* = 457)			
Nascimbeni et al.	2002	1997–1999	Case–control	Italy	192	Sigmoid cancer: Mean 65.6 (range 39–95) years; Diverticular disease: Mean 63.2 (range 28–90) years; Controls: Mean 64.3 (range 33–92) years	Anthracene laxatives (*n* = 37)	Not reported	Not reported	Colorectal cancer, melanosis coli
							No laxatives (*n* = 155)			
Charlton et al.	2013	2000–2009	Cohort with a case–control nested design	United Kingdom	33,138	Males: 73.0 (*SD* 10.9) years; Females: 74.9 (*SD* 12.2) years	Non‐macrogol only (*n* = 27,748)	Not reported	Not reported	Colorectal cancer
							Macrogol after other (*n* = 2,562)			
							Macrogol only (*n* = 1999)			
							Macrogol before other (*n* = 829)			

### 3.2 Risk of CRC in AQ laxatives users versus “other laxative” and “no laxative” users

**FIGURE 2:**
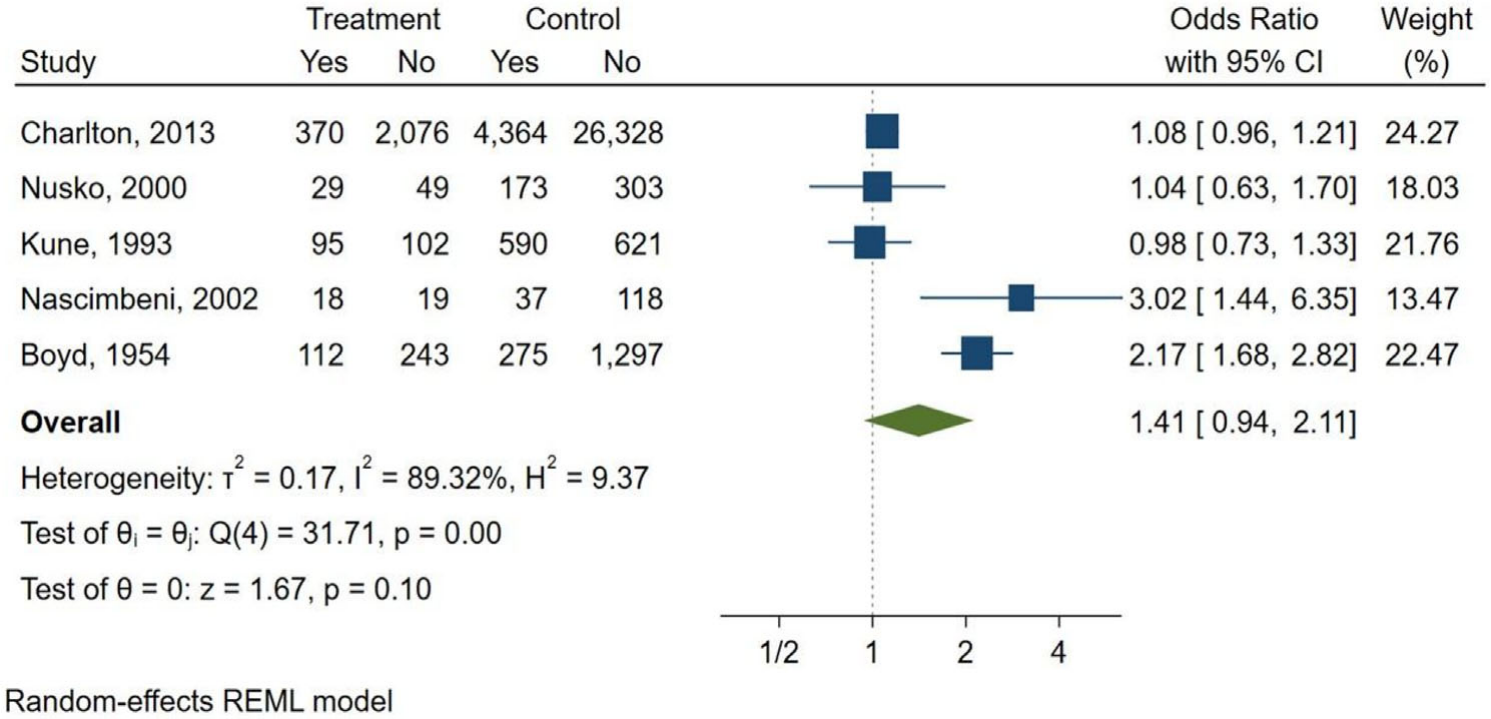
History of AQ laxatives use (AQ laxatives vs “other” or “no laxatives”) and risk of CRC

**FIGURE 3:**
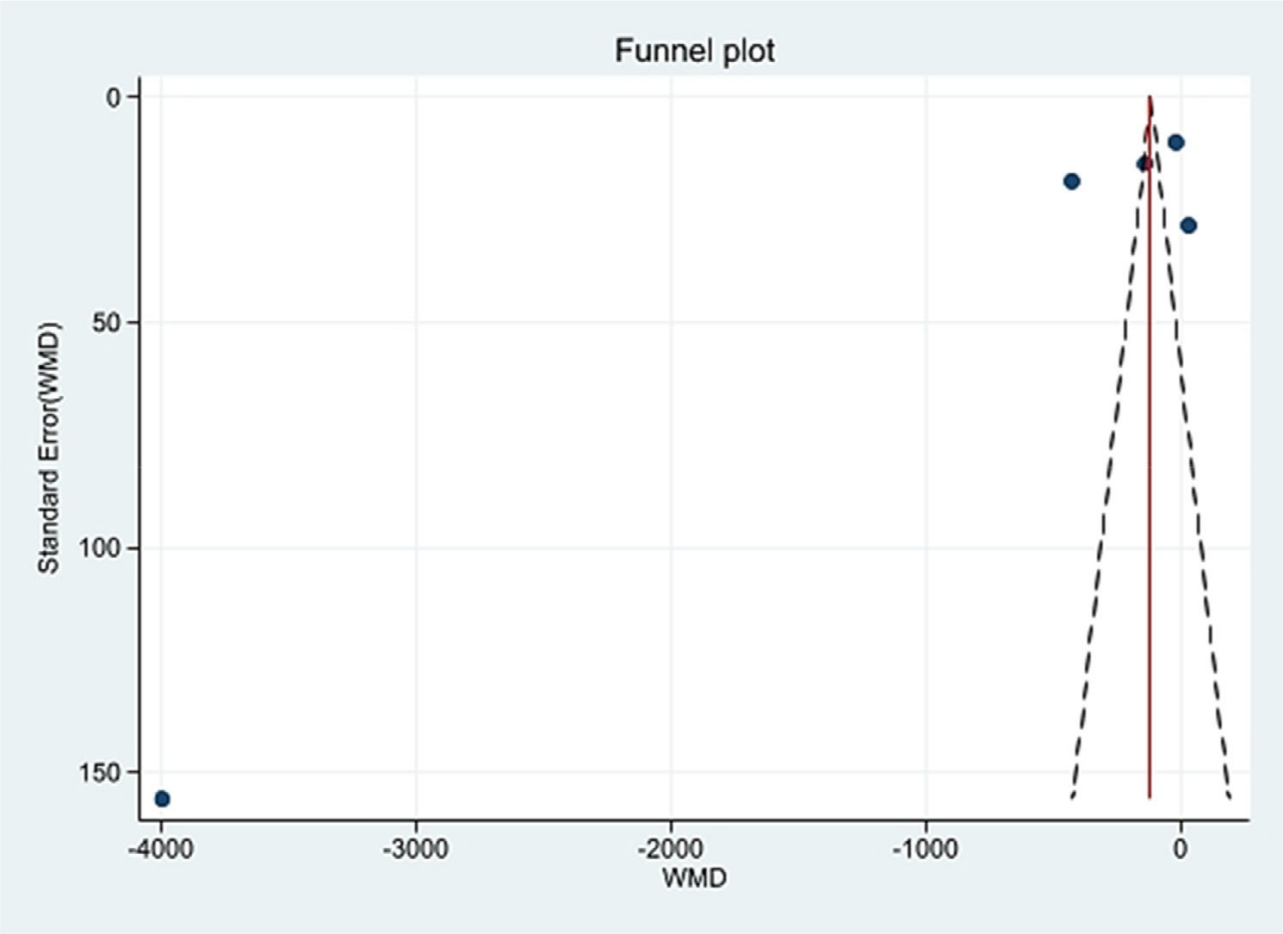
Funnel plot

**FIGURE 4:**
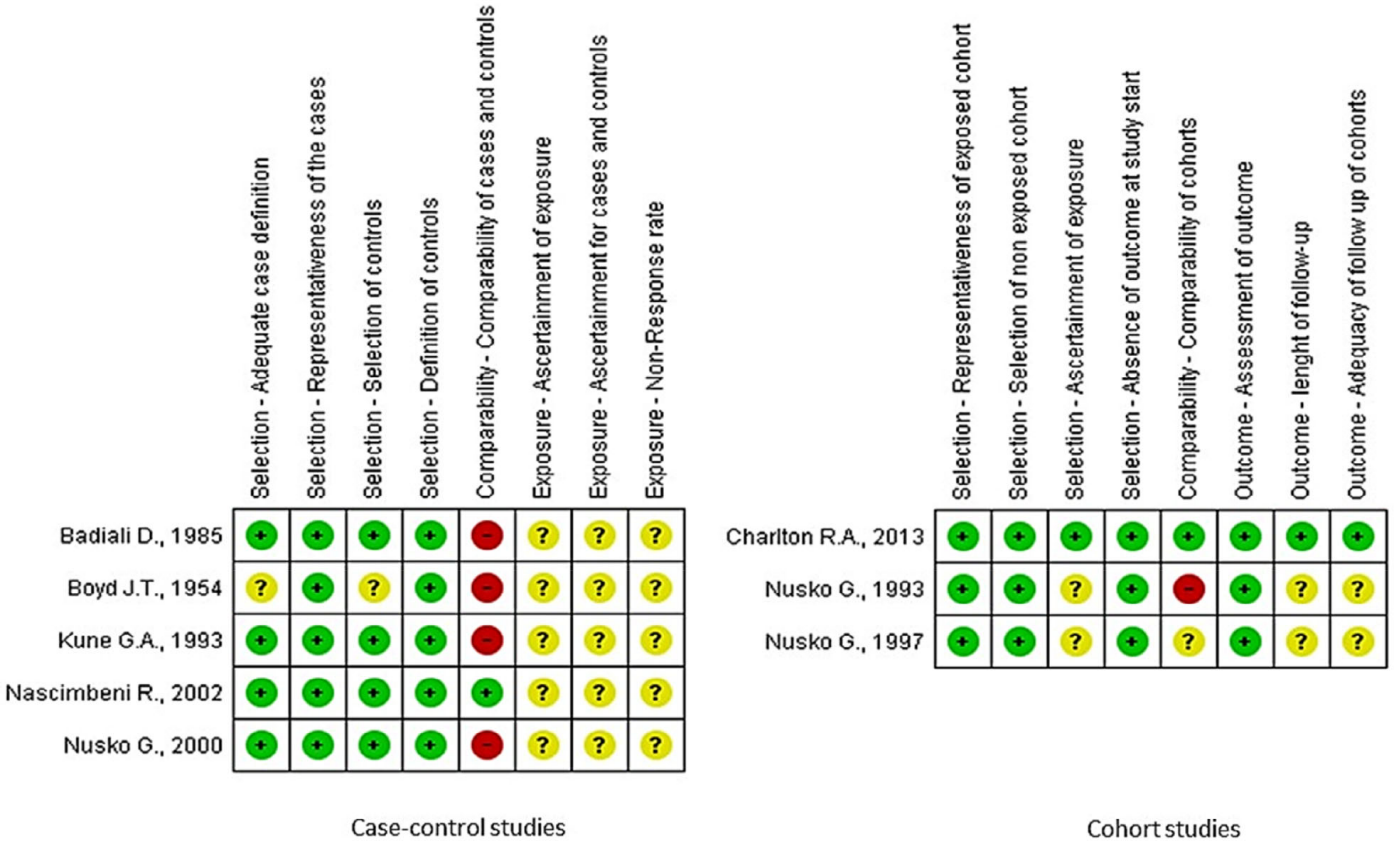
Quality assessment of included studies

Five studies (Boyd & Doll, 1954; Charlton et al., 2013; Kune, 1993; Nascimbeni et al., 2002; Nusko et al., 2000) were included in the meta‐analysis (Table 2), while three studies (Badiali et al., 1985; Nusko et al., 1993, 1997) were excluded due to the lack of information on AQ laxative use. In the included studies, the percentage of patients with a diagnosis of CRC ranged from 9.9 to 54%. In total, 6,063 patients had CRC (624 AQ laxative users; 5,439 “other laxatives” and “no laxative” users) and 31,156 patients did not have CRC (2,489 AQ laxative users; 28,667 “other laxatives” and “no laxative” users). When analysing all 5 studies using a random effects model, a history of AQ laxative use (any dosage and any treatment length) was associated with a non‐statistically significant increased risk of developing CRC (OR: 1.41; 95% CI: 0.94–2.11) compared to “other laxatives” and “no laxative” use (Figure 2). The funnel plot was examined visually and revealed major asymmetry (Figure 3). Based on visual analysis of the funnel plot, the study by Nusko et al. (2000) appeared to be an outlier. Removing this study did not significantly change risk estimates (OR: 1.51; 95% CI: 0.97–2.34) (*data not shown*). Only one study (Charlton et al., 2013) was judged at low risk of bias for each domain of the Newcastle‐Ottawa Scale (Figure 4). Selection of cases and controls was judged at low or unclear risk of bias across almost all studies, while comparability represented the domain at high risk of bias in the majority of the included studies.

**TABLE 2:** Full‐text manuscripts included in the meta‐analysis of colorectal cancer and anthracene laxatives use (*quantitative evidence*)

Study	Publication year	Mean age	Number of treated patients	Treatments	Outcome	Number of events (%)
Boyd and Doll	1954	Not reported	355	Anthracene laxatives	Cancer of large bowel	112 (31.5)
			774	Other laxatives		196 (25.3)
			798	No laxatives		79 (9.9)
Kune et al.	1993	65.0 years	197	Anthracene laxatives	Colorectal cancer	95 (48.2)
			127	Other laxatives		69 (54.3)
			1,084	No laxatives		521 (48.1)
Nusko et al.	2000	58.5 years	78	Anthracene laxatives	Colorectal neoplasm	29 (37.2)
			19	Other laxatives		7 (36.8)
			457	No laxatives		166 (36.3)
Nascimbeni et al.	2002	64.4 years	37	Anthracene laxatives	Sigmoid cancer	18 (48.6)
			155	No laxatives		37 (23.9)
Charlton et al.	2013	73.9 years	2,446	Dantron	Colorectal cancer	370 (15.1)
			30,692	Other laxatives		4,364 (14.2)

### 3.3 Subgroup analyses

**FIGURE 5:**
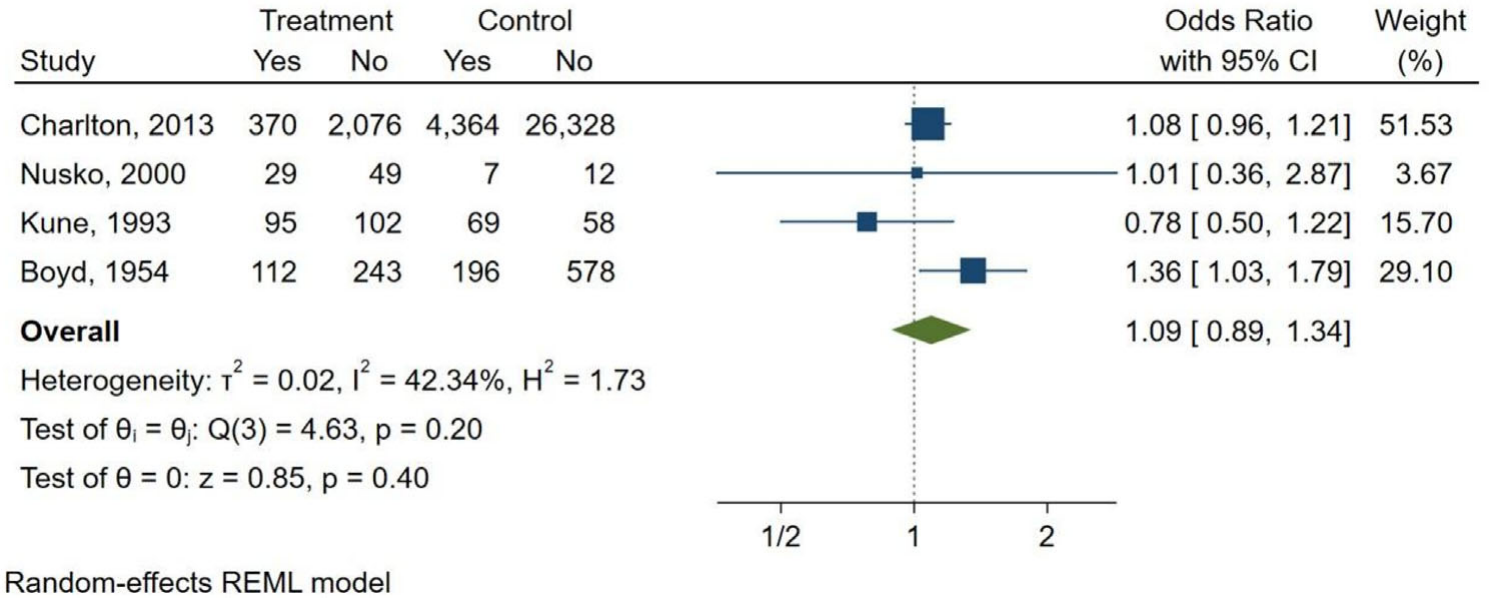
History of AQ laxatives use versus “other laxatives” and risk of CRC

**FIGURE 6:**
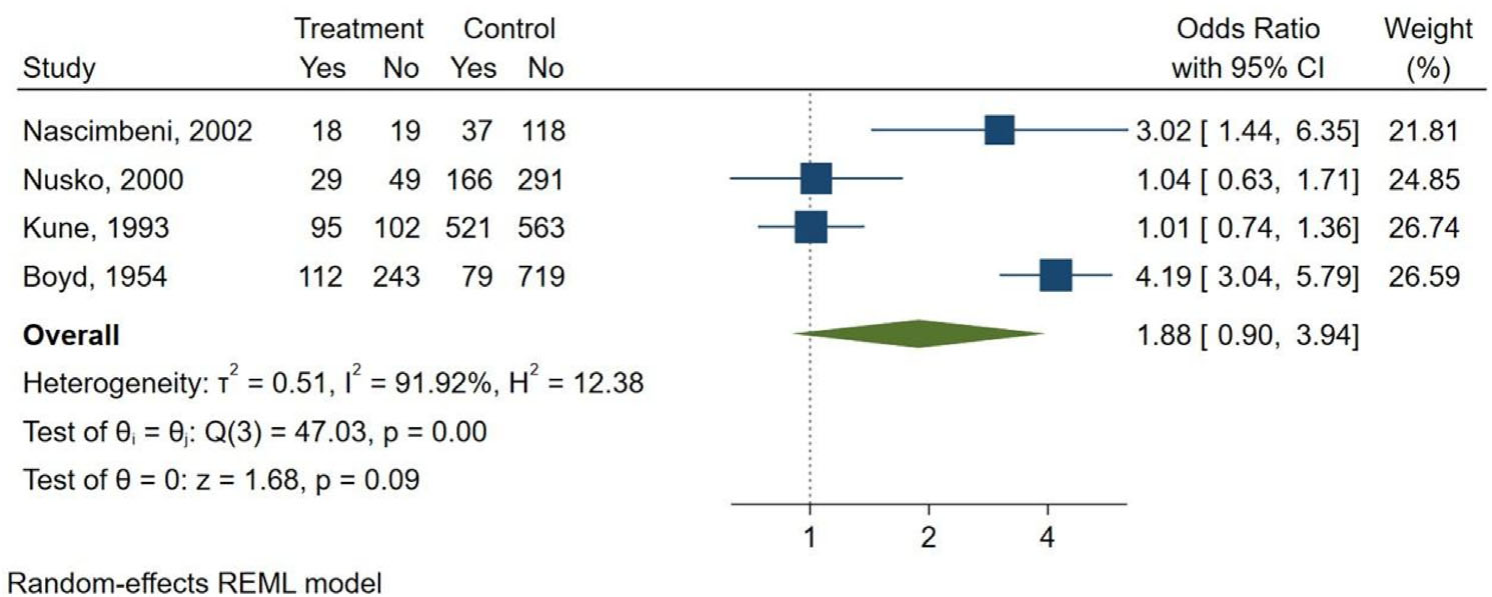
History of AQ laxatives use versus “no laxatives” and risk of CRC

Three studies compared AQ laxatives use to both “other laxatives” and “no laxative” use (Boyd & Doll, 1954; Kune, 1993; Nusko et al., 2000). Furthermore, one study compared AQ laxatives use versus “other laxatives” (Charlton et al., 2013), and one study versus “no laxative” use (Nascimbeni et al., 2002), respectively. There was a non‐statistically significant increase in the risk of CRC seen when comparing AQ laxatives versus “other laxatives” use (OR: 1.09; 95% CI: 0.89–1.34) (Figure 5 **)** and AQ laxatives versus “no laxatives” use (OR: 1.88; 95% CI: 0.90–3.94) (Figure 6). No other subgroup analyses could be performed, such as those based on the duration of use of the AQs laxative (>2 weeks), as none of the included studies reported complete data on the duration of treatment.

## 4 DISCUSSION

The European Commission recently prohibited the use of all preparations based on *Aloe* spp., as well as those containing emodin and aloe‐emodin, and placed under Union scrutiny the use of *Rheum*, *Cassia*, and *Rhamnus* and their preparations in food supplements, following the risk of harmful effects on health associated with their use (European Commission, 2006). In this scenario, our systematic review and meta‐analysis contributed to estimate the aforementioned overall risk (CRC and/or MC), although a small number of studies were included.

Considering MC, the role of AQ laxatives in its development could not be assessed. In fact, the association between AQ laxatives use and MC is mainly reported in the literature as anecdotal (i.e., case report, case series, endoscopy studies, etc.) description (Younes et al., 2018). Moreover, most of observational evidence incorrectly considers MC as a *proxy* of long‐term use of laxatives, specific for AQ derivatives (Kassim et al., 2020; Siegers, Von Hertzberg‐Lottin, Otte, & Schneider, 1993), but their use is only one of the factor involved in its aetiology (Yang et al., 2020). Actually, other causes such as irritable bowel syndrome, inflammatory bowel disease, colonic neoplasms, hyperplastic polyps, the use of nonsteroidal anti‐inflammatory drugs, vitamin E deficiency, the intake of unsaturated fatty acids, environmental factors, family history, psoriasis and Rett syndrome, feces stasis, and obstruction of colon and rectum could also contribute to the onset of MC (Yang et al., 2020). In this context, our results emphasise the need to evaluate the association between AQ laxatives and MC through large studies, with a proper follow‐up, a clear evaluation of comorbidities and other risk factors for MC onset, and a defined period of exposure. Noteworthy, AQ laxatives are known to be widely used as over‐the‐counter medications or self‐prescribed herbal products (Vitalone et al., 2012), making the assessment of the effective exposure to these products very difficult.

As reported in EFSA scientific opinion (Younes et al., 2018), previous epidemiological evaluations suggested an increased risk for CRC associated with the general use of laxatives (Sonnenberg & Muller, 1993), some of which contain AQ derivatives (Nusko et al., 1993; Siegers et al., 1993). To the best of our knowledge, this is the first systematic review and meta‐analysis investigating the role of AQ laxatives alone on the risk of gastrointestinal neoplasia. Focusing on studies only reporting AQ laxatives, our meta‐analysis showed that their intake was associated with a trend towards the development of CRC compared to “other” and “no laxatives” use. Actually, all studies had methodological limitations such as the use of a retrospective design, small sample size, and lack of adjustment for potential confounding factors. Moreover, no studies reported information on dosage and length of treatment with AQ laxatives. Anyway, our results may not be inconclusive (Alderson, 2003; Guyatt et al., 2011), especially from a clinical point of view.

Our study has several strengths. This is the first systematic review and meta‐analysis studying the association between AQ laxatives use and CRC. The strength of this analysis is bolstered by the large sample size of 41,873 patients. As can be seen from the risk of bias assessment, besides all of included studies are retrospective, they were judged at low risk of bias for cases and controls selection, and the length of follow‐up was sufficient to assess with certainty the outcomes of interest. There are also some limitations. First, heterogeneity between studies was high. Second, baseline information regarding cumulative AQ laxatives dose and length of treatment was unavailable in all studies. Considering the included studies, it was not possible to evaluate other clinically relevant confounding variables, such as patients' alimentary behaviour and constipation severity. Moreover, the high risk of bias for comparability and exposure may have influenced the results of this analysis, particularly for case–control studies. Given these limitations, our study provides the best risk estimate currently available for counselling patients undergoing AQ laxatives use.

In response to the EFSA considerations (Younes et al., 2018), since a history of AQ laxative use may be associated with an increased risk of CRC, it should be made mandatory to insert specific warnings on the labelling of food supplements containing AQ derivatives used as laxative, for example: (1) “This product is only recommended for the short‐term treatment of occasional constipation”; (2) “In case of previous diagnosis or family history of CRC the use of this product is not recommended”; (3) “Use this product only until intestinal function is restored. Otherwise, contact your doctor or pharmacist”; (4) “This product can induce the loss of electrolytes and therefore should avoided by patients receiving certain drugs (i.e., diuretics, digoxin, etc.)”; and (5) “This product increases intestinal motility, consequently can reduce the absorption of drugs administered concomitantly.” These warnings could be helpful especially for those individuals using AQ derivatives still on the market but currently under monitoring (i.e., *Rheum*, *Cassia*, and *Rhamnus*) (European Commission, 2021). The aforementioned warnings and contraindications could improve the appropriateness of both prescription and use of AQ derivatives, in particular those used as laxatives. Their use could also be applied to the labels of galenic preparations. For medicinal products containing AQ laxatives marketed in Italy, their summary of product characteristics already reports specific warnings and precautions, aimed to avoid an inappropriate use of these products.

## 5 CONCLUSIONS

In summary, qualitative and quantitative synthesis of observational studies does not show that AQ laxatives are associated with CRC development. Noteworthy, more extensive and high quality population studies are needed to collect information on the influence of AQ laxatives dosage and long‐term use as risk factors for the onset of this relevant disease. The theoretical risk of CRC should be considered for patients with other known risk factors for CRC. To date, it is not possible to associate the use of AQ laxatives with the development of CRC. However, the trend towards an increased risk of CRC provides a strong indication for investigating this issue through pharmaco‐ and phytovigilance systems in the next future.

In conclusion, this study may contribute to enhancing public attention and to alert healthcare professionals (general practitioners and pharmacists) and customers regarding the potential risks associated with the misuse/abuse of AQ laxatives.

## CONFLICT OF INTEREST

The authors declare no conflict of interest.

## AUTHOR CONTRIBUTIONS

**Niccolò Lombardi**: Conceptualization, Methodology, Validation, Investigation, Formal analysis, Writing – Original Draft. **Giada Crescioli**: Conceptualization, Methodology, Validation, Investigation, Formal analysis, Writing – Original Draft. **Valentina Maggini**: Conceptualization, Methodology, Validation, Investigation, Formal analysis, Writing – Original Draft. **Raffaele Bellezza**: Data Curation, Visualization. **Iacopo Landi**: Data Curation, Visualization. **Alessandra Bettiol**: Formal analysis. **Francesca Menniti‐Ippolito**: Validation, Writing – Review & Editing, Supervision. **Ilaria Ippoliti**: Data Curation, Visualization, Supervision. **Gabriela Mazzanti**: Writing – Review & Editing, Supervision. **Annabella Vitalone**: Writing – Review & Editing, Supervision. **Eugenia Gallo**: Data Curation. **Francesco Sivelli**: Data Curation. **Francesco Sofi**: Validation, Writing – Review & Editing, Supervision. **Gian Franco Gensini**: Validation, Writing – Review & Editing, Supervision. **Alfredo Vannacci**: Validation, Writing – Review & Editing, Supervision. **Fabio Firenzuoli**: Project administration.

## Data Availability

The data that support the findings of this study are available from the corresponding author upon reasonable request.
